# Characteristics of Teenagers Presenting with Chest Pain after COVID-19 mRNA Vaccination

**DOI:** 10.3390/jcm12134421

**Published:** 2023-06-30

**Authors:** Chul Hwan Park, Juyeon Yang, Hye Sun Lee, Tae Hoon Kim, Lucy Youngmin Eun

**Affiliations:** 1 Department of Radiology and Research Institute of Radiological Science, Gangnam Severance Hospital, Yonsei University College of Medicine, Seoul 06273, Republic of Korea; 2 Biostatistics Collaboration Unit, Yonsei University College of Medicine, Seoul 03722, Republic of Korea; 3 Department of Pediatrics, Gangnam Severance Hospital, Yonsei University College of Medicine, Seoul 06273, Republic of Korea

**Keywords:** COVID-19, vaccination, mRNA, chest pain, myocarditis, echocardiography, cardiac MRI

## Abstract

In this study, we evaluated the clinical and radiological manifestations of teenagers presenting with chest pain after coronavirus disease 2019 (COVID-19) messenger RNA (mRNA) vaccination. We retrospectively enrolled 61 teenage patients, aged 13 to 19 years, who underwent echocardiography and cardiac magnetic resonance imaging (CMR) for chest pain after COVID-19 mRNA vaccination, from November 2021 to April 2022. Medical records, laboratory results, echocardiographic, and CMR findings were analyzed. The mean age of the participants was 14.4 ± 1.9 years, with a male:female ratio of 28:33. Among the sixty-one patients with chest pain after COVID-19 vaccination, only two (3.3%) were diagnosed as confirmed myocarditis, and almost all of them recovered with conservative treatments. However, on CMR, 24 (39.3%) presented with mild myocardial abnormalities; 22 (36.1%) showed myocardial edema, and 19 (31.1%) were found to have a myocardial injury. Multivariate logistic analyses revealed that older age and female sex were significantly associated with myocardial abnormalities. In teenagers who present with chest pain after COVID-19 mRNA vaccination, confirmed myocarditis is uncommon. However, myocardial abnormalities on CMR might occur frequently, and females in their late teens might be more vulnerable to myocardial abnormalities.

## 1. Introduction

During the coronavirus disease 2019 (COVID-19) pandemic, expedited efforts to develop safe and effective vaccines led to the development of novel messenger RNA (mRNA)-based vaccines for severe acute respiratory syndrome coronavirus 2 (SARS-CoV-2) [1]. The mRNA vaccines have been distributed worldwide to mitigate SARS-CoV-2 infection, and the eligibility to receive the vaccine has been expanded to teenagers in high schools and middle schools above the age of 12 years [2]. However, concerns regarding the adverse effects of these vaccines have also increased [3]. Frequent adverse effects are usually mild, including injection site pain/swelling, fatigue, headache, fever, chills, and nausea [4]. However, acute myocarditis can occur, especially in young male adolescents, after the second vaccination [5]. Recently, COVID-19-related myocarditis has been an important clinical issue, and its pathophysiology is thought to be a combination of direct viral injury and cardiac damage due to the host immune response [6,7,8,9,10].

Among the various adverse symptoms, chest pain is a major symptom of acute myocarditis [11,12] and accurate stratification of patients with chest pain after mRNA vaccination is mandatory [13]. However, the clinical significance and stratification schemes for teenagers with chest pain after mRNA vaccination for COVID-19 have not yet been established.

In this retrospective study, we aimed to evaluate the clinical and radiological manifestations of teenagers presenting with chest pain after COVID-19 mRNA vaccination.

## 2. Materials and Methods

### 2.1. Patients

**Figure 1:**
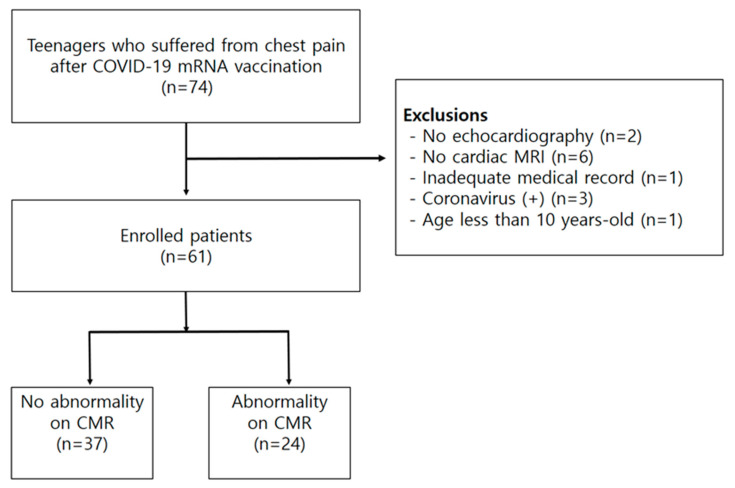
Patient selection flowchart in this study.

This was a retrospective observational study. Records of patients who underwent CMR at our institution between November 2021 and April 2022 were retrospectively reviewed. The inclusion criteria were as follows: (1) patients referred to our hospital because of chest pain after COVID-19 mRNA vaccination, (2) teenagers aged between 13 and 19 years, and (3) patients who underwent echocardiography and CMR within a 1-week interval. The exclusion criteria included the following: (1) a previous history of myocarditis or SARS-CoV-2 infection and (2) incomplete medical records. Figure 1 shows the patient selection flowchart.

This study was approved by the institutional review board of Gangnam Severance Hospital (approval number: 3-2021-0515). The requirement for written informed consent was waived owing to the retrospective nature of this study.

### 2.2. Laboratory Tests

Venous blood samples were collected from all patients. The lower detection limits for troponin I and troponin T assays were 0.012 and 0.003 mcg/L, respectively. In addition, basic, routine laboratory tests were performed, including complete blood cell count, routine chemistry, electrolyte levels, and iron profiles.

### 2.3. Transthoracic Echocardiography: Protocol

All patients underwent transthoracic echocardiography in the left lateral decubitus or supine position using commercially available echocardiography systems with the help of a 1.7–3.4 MHz transducer (E95, GE Vingmed Ultrasound, Horten, Norway), including two-dimensional, M-mode, color flow, conventional spectral Doppler, and tissue Doppler imaging (TDI). All data were acquired from parasternal, apical, and subcostal views. Three consecutive beats were stored in the cine-loop format, and the images were analyzed offline using dedicated software (EchoPAC, GE Healthcare, Chicago, IL, USA).

All examinations were performed in accordance with the American Society of Echocardiography. The measurements included in the analysis were as follows: left ventricular ejection fraction (LVEF, %), left ventricular end-diastolic dimension (mm), left ventricular end-systolic dimension (mm), interventricular septal thickness at diastole (mm), interventricular septal thickness at systole (mm), left ventricular posterior wall thickness at diastole (mm), left ventricular posterior wall thickness at systole (mm), mitral E (m/s), mitral A (m/s), E/A ratio, lateral E’ (m/s), lateral A’ (m/s), lateral S’ (m/s), lateral E/E’, septal E’ (m/s), septal A’ (m/s), septal S’ (m/s), septal E/E’, tricuspid E’ (m/s), tricuspid A’ (m/s), tricuspid S’ (m/s), tricuspid annular plane systolic excursion (TAPSE, mm), pulmonary artery velocity (m/s), and inferior vena cava diameter (mm). The mean value of more than three measurements obtained using echocardiography was used in the analysis.

### 2.4. Echocardiography: Analysis

Measurements were performed to determine whether they were within a reasonable range. This study defined myocardial injury or myocarditis as LVEF < 45%, noticeable segmental wall motion abnormality, abnormal findings on conventional Doppler spectral velocities, and advanced tissue Doppler velocities.

### 2.5. CMR: Protocol

All CMR examinations were performed using a 3.0-Tesla scanner (Magnetom Vida, Siemens Healthineers, Erlangen, Germany) with a 32-element phased-array coil. After cardiac localization, cine imaging with a steady-state free precession sequence, native T1 mapping using a modified look-locker inversion recovery (MOLLI) sequence with a 5(3)3 protocol, and T2 mapping using a T2-prepared balanced steady-state free precession sequence were performed. Ten minutes after intravenous injection of 0.2 mmol/kg of gadoterate dimeglumine (Dotarem, Guerbet, Villepinte, France), T1-weighted inversion recovery segmented breath–hold late gadolinium enhancement (LGE) imaging was performed with magnitude- and phase-sensitive inversion recovery reconstruction. The optimal inversion time was selected using an inversion time scout. Subsequently, post-contrast T1 mapping was performed using the MOLLI sequence with a 4(1)3(1)2 scheme. All CMR images were transferred to a picture archiving and communication system station (Centricity 4.0, GE Healthcare) and commercially available CMR-dedicated software (CVI42, Circle Cardiovascular Imaging, Calgary, AB, Canada) for image analysis. Extracellular volume (ECV) maps were generated semi-automatically with native T1 map images and post-contrast T1 map images.

### 2.6. CMR: Analysis

Two radiologists (with more than ten years of experience in cardiothoracic radiology) who were blinded to the clinical information, including the patient’s symptoms and laboratory data, independently reviewed the CMR images. The radiologists evaluated the CMR findings using the 2018 Lake–Louise criteria (LLC) [14]. Functional parameters, including LV end-diastolic volume (LVEDV), LV end-systolic volume (LVESV), LV stroke volume (LVSV), and LVEF, were acquired using cine imaging. The obtained functional parameters were normalized using the patient’s body surface area (BSA) and defined as LVEDVi, LVESVi, and LVSVi. Pericardial effusion was defined as a pericardial fluid collection at a depth of >4 mm.

Myocardial edema was evaluated using T2-based images (T2 maps), and a prolonged T2-relaxation time was defined as T2 values > 40 ms. Myocardial injury was assessed using T1-based imaging, including native T1 maps, ECV maps, and LGE imaging. Prolonged T1-relaxation time and ECV fraction were defined as T1 values > 1230 ms and ECV fractions higher than 28%, respectively [15].

### 2.7. Statistical Analyses

The distribution of data was evaluated using the Shapiro–Wilk test. Continuous variables are expressed as means ± standard deviations or medians (interquartile ranges), according to the results of the normality test. Categorical variables are expressed as frequencies (percentages). An independent two-sample *t*-test or Mann–Whitney U test was used to compare demographic, echocardiographic, or CMR parameters between the groups. Interobserver agreements measuring T2 values, T1 values, and ECV fractions were evaluated using the intraclass correlation coefficient (ICC). Univariate logistic regression analysis was used to assess the association between myocardial changes and patient demographics, laboratory examinations, and echocardiographic parameters. Multivariable logistic regression was used to identify independent factors, including demographics, laboratory test results, and echocardiographic parameters for myocardial changes in the MR parameters. For this analysis, variables from the univariable analysis with *p* < 0.1 were entered. If there was a significant correlation between the independent variables, only one variable was entered into the model to avoid multicollinearity. Statistical significance was set at *p* < 0.05. All statistical analyses were performed using SAS (version 9.4, SAS Inc., Cary, NC, USA).

## 3. Results

### 3.1. Basic Patient Characteristics

In total, 61 patients were enrolled in this study. The mean age of the participants was 14.4 ± 1.9 years, with a male:female ratio of 28:33. The mean physical characteristics included the following: height, 164.1 ± 9.4 cm; weight, 59.0 ± 14.7 kg; body mass index (BMI), 21.7 ± 3.9 kg/m^2^; and BSA, 1.64 ± 0.23 m^2^. None of the patients were presented with baseline comorbidities, including asthma, diabetes mellitus, or autoimmune diseases. Detailed baseline characteristics of the enrolled patients are summarized in Table 1.

### 3.2. Clinical Presentation and Laboratory Results

**Figure 2:**
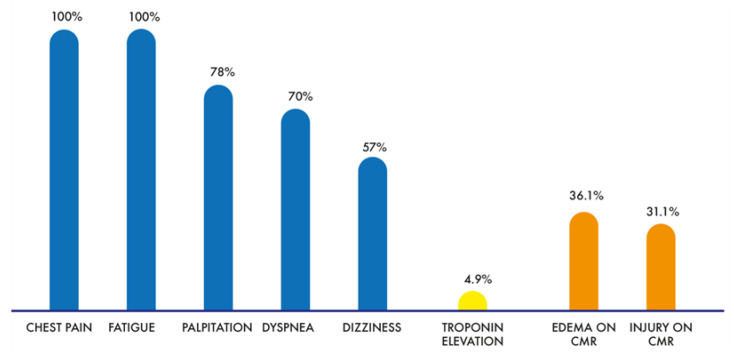
Symptoms and results of the diagnostic studies.

According to the government’s policy, all patients partook in the COVID-19 mRNA vaccination program and were administered the Pfizer–BioNTech vaccine. All patients included in this study presented with chest pain (100%); 26 (42.6%) and 35 (57.4%) patients developed chest pain after the first and second vaccinations, respectively. Other symptoms included fatigue (100%), palpitations (78%), dyspnea (70%), dizziness (57%), headache (45%), and epigastric pain (8%). Electrocardiography (ECG) revealed a normal sinus rhythm in 47 patients (77%), whereas 14 patients (23%) presented with various abnormal features, including incomplete right bundle branch block, incomplete left bundle branch block, premature ventricular contraction, sinus bradycardia, T-wave inversion, and borderline QT prolongation. However, none of the patients showed ST elevation, a typical sign of myocarditis. The mean hemoglobin (Hb) level was 13.5 ± 1.1 g/dL, and the hematocrit level was 41.7 ± 3.4%. Of the sixty-one patients, three (4.9%) had elevated serum troponin T (ref: 0.003–0.014 mcg/L) and troponin I (0.012–0.034 mcg/L) levels, above the upper limit of the reference level. The levels of C-reactive protein, creatine kinase MB fraction, and N-terminal pro-B-type natriuretic peptide were not statistically different between groups (all *p* > 0.05) (Table 2, Figure 2).

### 3.3. Echocardiographic Findings

We used conventional Doppler echocardiography to evaluate LV mitral inflow, as well as TDI to quantitatively assess LV and right ventricular (RV) systolic and diastolic myocardial movements. The mean LVEF of participants was 67.50 ± 5.48%. All 61 patients had LVEF ≥ 55%; thus, regardless of myocardial edema or injury noted on CMR, no patient showed LVEF < 45%. Compared to patients with no abnormalities identified in CMR, there were no statistical differences in echocardiographic measurements in patients with myocardial edema or injury, including tissue Doppler velocity. Additionally, the findings representative of myocardial deterioration—lateral E/E and septal E/E—showed no difference in patients with myocardial edema or injury. RV systolic functional measurement using TAPSE also indicated no difference in patients with myocardial edema or injury (Table 3).

### 3.4. CMR Findings

The median time between vaccination and CMR examination was seven days (1Q: 4.0 days; 3Q: 21.5 days). Based on the modified LLC, 22 patients (36.1%) showed abnormalities on T2-based imaging and were diagnosed with myocardial edema. Nineteen patients (31.1%) showed abnormalities on T1-based imaging and were diagnosed with myocardial injury. Seventeen patients (27.8%) showed simultaneous myocardial edema and injury, and twenty-four (39.3%) presented with either myocardial edema or injury. Thirty-seven patients (60.7%) showed no abnormalities on T1- and T2-based imaging. Two of the three patients with elevated serum troponin levels had myocardial edema and injury; however, the third patient showed negative findings.

On cine imaging, mean LVEDVi, LVESVi, SVi, and LV mass index for the 61 participants were 74.6 ± 12.2 mL/m^2^, 26.7 ± 6.9 mL/m^2^, 47.9 ± 8.3 mL/m^2^, and 56.4 ± 8.4 g/m^2^, respectively. The LVEF was greater than 55% in all patients, with a mean LVEF of 64.6% ± 5.5%. The LV mass index was significantly lower in patients with myocardial edema or injury than those without myocardial edema or injury (*p* = 0.0014). However, LVEDVi, LVESVi, SVi, and LVEF did not differ between patients with and without myocardial abnormalities (all *p* > 0.05).

**Figure 3:**
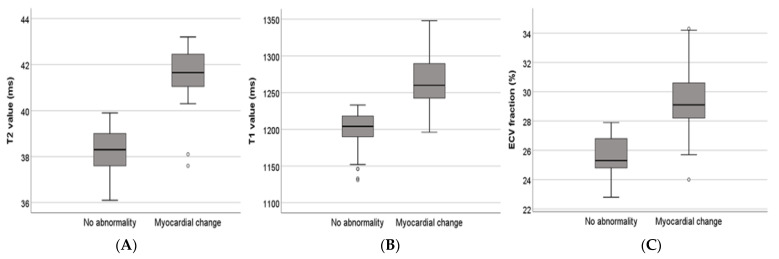
Comparisons of CMR findings between patients with or without myocardial abnormalities. Patients with myocardial changes (edema or injury) show higher T2 values (**A**), native T1 values (**B**), and ECV fractions (**C**), compared to the participants with negative CMR findings (all *p* < 0.05). CMR: cardiac magnetic resonance imaging; ECV: extracellular volume.

In all patients, the mean T2 value, native T1 value, and ECV fraction of the LV myocardium were 39.5 ± 2.0 ms, 1225.0 ± 45.7 ms, and 27.1 ± 2.6%, respectively. The mean T2 value of patients with myocardial edema was 41.9 ± 0.8 ms, which was significantly higher than that of patients without myocardial edema (38.2 ± 1.0 ms). The mean T1 value and ECV fraction of patients with myocardial injury were 1265.9 ± 37.6 ms and 30.2 ± 1.9%, respectively, which were significantly higher than those of patients without myocardial injury (1206.5 ± 36.2 ms and 25.7 ± 1.3%, respectively). Patients with myocardial abnormalities (edema or injury) showed higher T2 values, native T1 values, and ECV fractions than did the participants with negative CMR findings (*p* < 0.001) (Table 4, Figure 3). The interobserver agreements for measuring T2 values, T1 values, and ECV fraction were 0.887, 0.929, and 0.911, respectively.

### 3.5. Logistic Regression Analyses for Myocardial Abnormalities on CMR

Univariable logistic regression analyses revealed that myocardial abnormalities (myocardial edema or injury) were significantly associated with the female sex and lower weight, BMI, and BSA values. Among the laboratory parameters, Hb and hematocrit levels were associated with myocardial abnormalities on CMR. Echocardiographic parameters were not associated with any myocardial abnormalities (Table 5).

In the multivariable logistic regression analyses, a myocardial abnormality was significantly associated with age and female sex (Table 6). BSA showed multicollinearity with BMI, and the female sex showed significant multicollinearity with Hb level. BSA and female sex were included in the model instead of BMI and Hb to avoid multicollinearity.

### 3.6. Clinical Courses of Patients

The chest pain developed within 72 h to 7 days from the vaccination. Then the chest pain continued for approximately 7 to 10 days. All the patients were treated conservatively for 3–7 days to achieve symptomatic relief of chest pain. After this treatment period, patients presented reduced symptoms and favorable clinical courses.

## 4. Discussion

This study evaluated the clinical and radiological manifestations of teenagers with chest pain after COVID-19 mRNA vaccination and analyzed the risk factors for myocardial abnormalities. Among the 61 patients enrolled, only two cases were confirmed as myocarditis. However, myocardial abnormalities were detected on CMR images in 24 (39.3%) of the 61 patients. In addition, we found that myocardial abnormalities were significantly associated with older age and the female sex.

**Figure 4:**
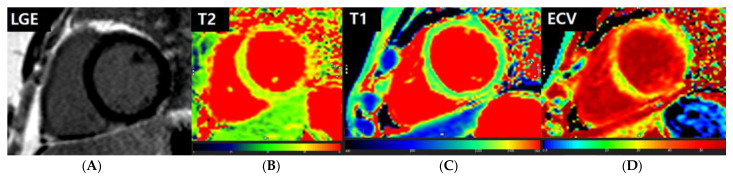
Representative images of a patient with probable myocarditis. A 16-year-old female patient presented with chest pain after COVID-19 mRNA vaccination. The patient’s troponin level was not elevated. On CMR, there is no abnormal hyperenhancement in the LV on LGE images (**A**). However, the T2 map shows diffusely increased T2 values of the myocardium, with a mean value of 42.6 ms (**B**). The native T1 map shows diffusely increased native T1 values of the myocardium, with a mean value of 1342 ms (**C**). The extracellular volume (ECV) map indicates an elevated ECV fraction of 34.2% (**D**). Based on the CMR findings, this patient was declared as having probable myocarditis.

**Figure 5:**
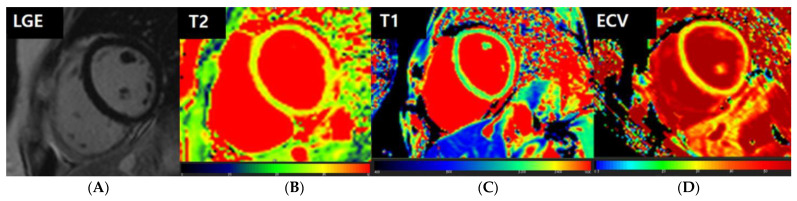
Representative images of a patient with confirmed myocarditis. An 18-year-old female patient presented with chest pain after COVID-19 mRNA vaccination. The patient’s troponin T level was elevated to 0.035 mg/dL. On CMR, there is no abnormal hyperenhancement in the LV on LGE images (**A**). However, the T2 map shows diffusely increased T2 values of the myocardium, up to 45.2 ms (**B**). The native T1 map shows diffusely increased native T1 values of the myocardium, with a mean value of 1276 ms (**C**). The extracellular volume (ECV) map indicates an elevated ECV fraction of 32.7% (**D**). Based on the CDC case definition, the patient was diagnosed with myocarditis.

Since chest pain is a significant symptom of myocarditis reported after mRNA vaccination [16], adolescents presenting with chest pain after vaccination should be carefully examined. The initial evaluation of COVID-19-vaccine-associated chest pain usually includes measuring troponin levels, electrocardiography, and echocardiography [10,17,18,19,20]. However, CMR could also play an important role in the evaluation of myocardial abnormalities [14,21,22,23]. Herein, all enrolled patients presented with chest pain. Even with normal ECG or normal troponin levels, CMR was performed to rule out potential myocardial injury if the patients complained of continuous chest pain. Parametric mapping techniques might offer quantitative data reflecting myocardial tissue characteristics, such as myocardial edema and injury [15,24], and updated LLC can offer improved diagnostic performance when evaluating myocarditis using T1- and T2-based imaging [23]. We identified myocardial edema or injury in 39.3% of the patients using CMR. Based on the updated LLC [14] and Centers for Disease Control and Prevention (CDC) Case Definitions [25,26], we diagnosed eighteen patients (29.5%) with probable myocarditis (seventeen with positive CMR findings and one with elevated troponin level) (Figure 4), while two cases met the description for confirmed myocarditis with elevated troponin levels and corresponding CMR findings (Figure 5).

In this study, myocardial abnormalities accompanied by chest pain tended to be less severe than reported myocarditis after vaccination. There was no obvious LGE in any patient, and the elevation of T1 values, T2 values, and ECV fraction was marginal. On the contrary, previous studies reported that patients with myocarditis after vaccination usually showed LGE, obviously increased T1 and T2 values, and ECV fraction [27,28,29,30]. Shiyovich et al. [31] reported that post-vaccination myocarditis with increased T1 values occurred in 46% of patients and LGE in 87%. In addition, the patient characteristics in this study differ from those of previous studies on myocarditis, which reported the occurrence of myocarditis a few days after the second dose in male adolescents [2,5,32]. Here, the duration of symptoms was prolonged, and myocardial abnormalities were associated with older ages and female sex. Several characteristics that showed a positive association with myocardial changes in univariable analysis, such as lower BMI, BSA, and Hb values, might be common features of female teens. These findings suggest that mild myocardial abnormalities, even without myocarditis, could occur after vaccination in female adolescents presenting with chest pain.

Concerning the CMR findings, echocardiography did not reveal any definite myocardial abnormalities; none of our patients had LVEF < 45%. Instead, LVEF was >55% in all 61 patients, indicating proper contractile function. We assume that a subtle diastolic alteration may occur after mRNA vaccination, which could be recognized on echocardiography as probable myocarditis, even with reasonable ranges of mitral E, without systolic dysfunction. However, echocardiographic information is insufficient to confirm mild myocardial abnormalities without a CMR examination.

This study has several limitations. First, this was a single-center retrospective study with a small sample size. Second, although the enrolled patients presented with chest pain after vaccination, the myocardial change on CMR was not pathologically proven. Long-term surveillance should be conducted to determine the natural course of myocardial edema or injury associated with COVID-19 mRNA vaccines. This can help to identify characteristics of myocardial changes on CMR. However, many teenagers and children continue to being exposed to SARS-CoV-2, and COVID-19 vaccines are highly recommended for every teenager and child that is eligible. Therefore, our study will be a helpful guide for teenagers presenting with chest pain after vaccination.

## 5. Conclusions

In teenagers who present with chest pain after COVID-19 mRNA vaccination, myocarditis is uncommon, and the clinical course is usually benign. However, myocardial abnormalities on CMR might be of frequent occurrences. Females in their late teens might be more vulnerable to myocardial abnormalities, with continuous chest pain one week after vaccination.

## Figures and Tables

**Table 1:** Basic demographic characteristics of the participants.

	All Patients (n = 61)	Myocardial Edema on CMR			Myocardial Injury on CMR			Myocardial Edema or Injury (Abnormality) on CMR		
		(+) (n = 22)	(−) (n = 39)	*p*-Value	(+) (n = 19)	(−) (n = 42)	*p*-Value	(+) (n = 24)	(−) (n = 37)	*p*-Value
Age (years)	14.4 ± 1.9	15.0 ± 2.1	14.1 ± 1.8	0.0915	15.1 ± 2.2	14.2 ± 1.8	0.0983	15.0 ± 2.0	14.1 ± 1.8	0.0699
Male:Female	28:33	3:19	25:14	0.0001	3:16	25:17	0.0015	3:21	25:12	<0.0001
Height (cm)	164.1 ± 9.4	161.3 ± 7.7	165.7 ± 9.9	0.0736	160.6 ± 8.5	165.7 ± 9.4	0.0494	161.1 ± 7.6	166.1 ± 10.0	0.0429
Weight (kg)	59.0 ± 14.7	52.9 ± 11.9	62.5 ± 15.1	0.0135	54.1 ± 12.0	61.3 ± 15.4	0.0749	53.0 ± 11.5	63.0 ± 15.3	0.0082
BMI (kg/m^2^)	21.7 ± 3.9	20.2 ± 3.7	22.5 ± 3.9	0.0291	20.8 ± 3.6	22.1 ± 4.0	0.2464	20.3 ± 3.5	22.6 ± 3.9	0.0239
BSA (m^2^)	1.64 ± 0.23	1.54 ± 0.19	1.69 ± 0.24	0.0144	1.56 ± 0.20	1.68 ± 0.24	0.0629	1.54 ± 0.19	1.70 ± 0.24	0.0083

BMI: body mass index; BSA: body surface area; CMR: cardiac magnetic resonance imaging. Data are shown as mean ± standard deviation.

**Table 2:** Clinical presentations and laboratory findings.

	All Patients (n = 61)	Myocardial Edema on CMR			Myocardial Injury on CMR			Myocardial Edema or Injury (Abnormality) on CMR		
		(+) (n = 22)	(−) (n = 39)	*p*-Value	(+) (n = 19)	(−) (n = 42)	*p*-Value	(+) (n = 24)	(−) (n = 37)	*p*-Value
Vaccination (1st:2nd)	26:35	8:14	18:21	0.4578	7:12	19:23	0.5392	9:15	17:20	0.5146
Duration * (median, (1Q, 3Q))	7.0 (4.0, 16.0)	7.0 (3.0, 14.0)	7.0 (5.0, 22.0)	0.4117	7.0 (4.0, 16.0)	7.0 (4.0, 22.0)	0.6619	7.0 (3.0, 14.0)	7.0 (4.5, 19.5)	0.5636
CRP (mg/L)	2.8 ± 4.9	2.9 ± 3.7	2.8 ± 5.5	0.896	3.0 ± 3.8	2.8 ± 5.3	0.839	2.7 ± 3.5	2.9 ± 5.6	0.880
CK-MB (ng/mL)	0.95 ± 1.53	1.20 ± 2.12	0.81 ± 1.10	0.356	1.31 ± 2.35	0.80 ± 1.06	0.244	1.12 ± 2.04	0.84 ± 1.13	0.499
NTproBNP (pg/mL)	30.5 ± 31.5	33.2 ± 24.9	29.0 ± 34.7	0.624	33.4 ± 26.1	29.3 ± 33.5	0.661	31.9 ± 24.1	29.6 ± 35.5	0.784
Hemoglobin (g/dL)	13.5 ± 1.1	12.8 ± 0.9	13.9 ± 1.0	<0.0001	12.9 ± 0.9	13.8 ± 1.1	0.0017	12.8 ± 0.9	14.0 ± 1.0	<0.0001
Hematocrit (%)	41.7 ± 3.4	39.6 ± 2.9	42.9 ± 3.1	0.0001	39.7 ± 2.8	42.6 ± 3.2	0.001	39.6 ± 2.8	43.1 ± 3.0	<0.0001
Serum iron (mcg/dL)	80.1 ± 40.7	76.5 ± 43.2	82.1 ± 39.7	0.6052	84.8 ± 43.8	77.9 ± 39.6	0.5434	79.0 ± 43.0	80.8 ± 39.7	0.8688
Iron saturation (%)	22.7 ± 11.8	22.6 ± 12.9	22.7 ± 11.3	0.9946	25.4 ± 12.8	21.4 ± 11.2	0.2266	23.1 ± 12.6	22.4 ± 11.4	0.8183
Ferritin (ng/mL)	51.6 ± 37.8	46.2 ± 37.2	54.7 ± 38.3	0.4039	50.7 ± 39.0	52.1 ± 37.7	0.8999	45.2 ± 36.5	55.8 ± 38.6	0.2896

* Days between vaccination and CMR examination; CK-MB: creatine kinase MB fraction; CMR: cardiac magnetic resonance imaging; CRP: C-reactive protein; NT-proBNP: N-terminal pro-B-type natriuretic peptide. Data are shown as mean ± standard deviation.

**Table 3:** Echocardiographic findings.

	All Patients (n = 61)	Myocardial Edema or Injury (Abnormality) on CMR		
		Abnormality (+) (n = 24)	Abnormality (−) (n = 37)	*p*-Value
LVEF (%)	67.50 ± 5.48	68.99 ± 4.49	66.54 ± 5.90	0.0879
LVEDD (mm)	47.30 ± 4.13	46.83 ± 4.15	47.60 ± 4.14	0.4852
LVESD (mm)	28.98 ± 4.76	28.61 ± 2.89	29.21 ± 5.69	0.5906
IVSd (mm)	7.09 ± 1.40	7.01 ± 1.22	7.15 ± 1.51	0.7184
IVSs (mm)	9.96 ± 1.83	9.90 ± 1.83	10.00 ± 1.85	0.8433
LVPWd (mm)	6.63 ± 1.30	6.66 ± 1.28	6.62 ± 1.33	0.9027
LVPWs (mm)	11.39 ± 2.45	11.49 ± 3.06	11.32 ± 2.00	0.8053
Mitral E (m/s)	1.022 ± 0.16	1.012 ± 0.19	1.029 ± 0.134	0.6652
Mitral A (m/s)	0.489 ± 0.118	0.485 ± 0.096	0.491 ± 0.131	0.8631
E/A	2.20 ± 0.53	2.13 ± 0.46	2.24 ± 0.57	0.4130
Lateral E′ (m/s)	0.179 ± 0.036	0.180 ± 0.045	0.178 ± 0.029	0.8155
Lateral A′ (m/s)	0.079 ± 0.071	0.092 ± 0.111	0.071 ± 0.018	0.3650
Lateral S′ (m/s)	0.129 ± 0.097	0.117 ± 0.026	0.137 ± 0.122	0.3369
Lateral E/E′	5.76 ± 1.23	5.50 ± 1.06	5.93 ± 1.31	0.1783
Septal E′ (m/s)	0.146 ± 0.024	0.146 ± 0.026	0.145 ± 0.023	0.9462
Septal A′ (m/s)	0.069 ± 0.083	0.057 ± 0.009	0.076 ± 0.106	0.2769
Septal S′ (m/s)	0.088 ± 0.016	0.089 ± 0.015	0.088 ± 0.017	0.8778
Septal E/E′	7.25 ± 1.63	7.27 ± 2.06	7.24 ± 1.32	0.9615
Tricuspid E′ (m/s)	0.158 ± 0.029	0.163 ± 0.032	0.156 ± 0.027	0.3684
Tricuspid A′ (m/s)	0.093 ± 0.026	0.091 ± 0.019	0.095 ± 0.030	0.5780
Tricuspid S′ (m/s)	0.136 ± 0.018	0.137 ± 0.016	0.136 ± 0.019	0.8509
TAPSE (mm)	22.88 ± 3.31	22.81 ± 3.37	22.91 ± 3.33	0.9137
PA velocity (m/s)	0.99 ± 0.18	0.99 ± 0.21	0.99 ± 0.17	0.9996
IVC diameter (mm)	18.68 ± 3.30	18.66 ± 3.14	18.68 ± 3.45	0.9811

CMR: cardiac magnetic resonance imaging; IVC: inferior vena cava; IVSd: interventricular septal thickness at diastole; IVSs: interventricular septal thickness at systole; LVEDD: left ventricular end-diastolic dimension; LVEF: left ventricular ejection fraction; LVESD: left ventricular end-systolic dimension; LVPWd: left ventricular posterior wall thickness at diastole; LVPWs: left posterior ventricular wall at end-diastole; PA: pulmonary artery; TAPSE: tricuspid annular plane systolic excursion. Data are shown as mean ± standard deviation.

**Table 4:** CMR findings.

	All Patients (n = 61)	Myocardial Edema on CMR			Myocardial Injury on CMR			Myocardial Edema or Injury (Abnormality) on CMR		
		(+) (n = 22)	(−) (n = 39)	*p*-Value	(+) (n = 19)	(−) (n = 42)	*p*-Value	(+) (n = 24)	(−) (n = 37)	*p*-Value
LVEDV (mL)	123.1 ± 31.1	112.5 ± 26.7	129.1 ± 32.1	0.0435	112.7 ± 27.9	127.8 ± 31.6	0.0777	112.3 ± 26.0	130.1 ± 32.4	0.0282
LVESV (mL)	44.1 ± 14.6	39.5 ± 11.0	46.7 ± 15.8	0.0656	39.5 ± 11.4	46.2 ± 15.5	0.0959	39.6 ± 10.8	47.0 ± 16.1	0.0369
SV (mL)	78.9 ± 19.8	73.0 ± 17.5	82.2 ± 20.4	0.0797	73.3 ± 18.5	81.4 ± 20.0	0.1369	72.7 ± 17.0	82.9 ± 20.6	0.05
LVEF (%)	64.6 ± 5.5	64.9 ± 3.8	64.5 ± 6.3	0.7584	65.0 ± 3.9	64.5 ± 6.1	0.718	64.8 ± 3.7	64.5 ± 6.5	0.8653
LV mass (g)	93.4 ± 24.0	81.2 ± 17.6	100.3 ± 24.4	0.0021	83.4 ± 18.6	98.0 ± 24.8	0.026	81.1 ± 17.6	101.4 ± 24.2	0.0008
LVEDVi (mL/m^2^)	74.6 ± 12.2	72.4 ± 12.4	75.8 ± 12.1	0.3085	71.8 ± 12.2	75.8 ± 12.2	0.2342	72.4 ± 12.0	76.0 ± 12.3	0.2606
LVESVi (mL/m^2^)	26.7 ± 6.9	25.5 ± 6.0	27.3 ± 7.3	0.3285	25.2 ± 5.8	27.3 ± 7.3	0.2599	25.6 ± 5.8	27.4 ± 7.5	0.3232
SVi (mL/m^2^)	47.9 ± 8.3	47.0 ± 7.8	48.4 ± 8.6	0.5261	46.7 ± 8.0	48.4 ± 8.4	0.446	46.8 ± 7.5	48.5 ± 8.8	0.4354
LV mass-i (g/m^2^)	56.4 ± 8.4	52.3 ± 7.2	58.6 ± 8.2	0.0036	53.2 ± 7.3	57.8 ± 8.5	0.0437	52.2 ± 7.3	59.0 ± 8.0	0.0014
LGE	None									
T2 value (ms)	39.5 ± 2.0	41.9 ± 0.8	38.2 ± 1.0	<0.0001	41.3 ± 1.4	38.7 ± 1.7	<0.0001	41.5 ± 1.4	38.2 ± 1.0	<0.0001
T1 value (ms)	1225.0 ± 45.7	1268.7 ± 37.5	1200.3 ± 28.1	<0.0001	1265.9 ± 37.6	1206.5 ± 36.2	<0.0001	1265.6 ± 37.6	1198.6 ± 27.6	<0.0001
ECV fraction (%)	27.1 ± 2.6	29.4 ± 2.5	25.8 ± 1.4	<0.0001	30.2 ± 1.9	25.7 ± 1.3	<0.0001	29.4 ± 2.4	25.6 ± 1.2	<0.0001

CMR: cardiac magnetic resonance imaging; ECV: extracellular volume; LGE: late gadolinium enhancement; LV mass: left ventricular mass; LV mass-i: left ventricular mass index; LVEDV: left ventricular end-diastolic volume; LVEDVi: left ventricular end-diastolic volume index; LVEF: left ventricular ejection fraction; LVESV: left ventricular end-systolic volume; LVESVi: left ventricular end-systolic volume index; SV: stroke volume; SVi: stroke volume index. Data are shown as mean ± standard deviation.

**Table 5:** Univariable logistic regression analyses of myocardial abnormalities on CMR.

	Myocardial Edema on CMR		Myocardial Injury on CMR		Myocardial Edema or Injury (Abnormality) on CMR	
	OR (95% CI)	*p*-Value	OR (95% CI)	*p*-Value	OR (95% CI)	*p*-Value
Demography						
Age (years)	1.314 (0.952–1.812)	0.0964	1.326 (0.946–1.859)	0.1013	1.334 (0.970–1.834)	0.0762
Sex (male:female)	11.310 (2.839–45.057)	0.0006	7.843 (1.976–31.128)	0.0034	14.583 (3.626–58.657)	0.0002
Height (cm)	0.947 (0.889–1.009)	0.0899	0.940 (0.880–1.005)	0.0680	0.939 (0.879–1.002)	0.0586
Weight (kg)	0.947 (0.904–0.992)	0.0209	0.962 (0.920–1.005)	0.0825	0.944 (0.902–0.989)	0.0145
BMI (kg/m^2^)	0.843 (0.718–0.989)	0.0365	0.915 (0.788–1.063)	0.2450	0.842 (0.719–0.984)	0.0310
BSA (m^2^)	0.037 (0.002–0.618)	0.0217	0.083 (0.006–1.234)	0.0707	0.030 (0.002–0.496)	0.0143
Duration	1.017 (0.985–1.051)	0.3020	1.025 (0.990–1.061)	0.1671	1.016 (0.984–1.049)	0.3383
Laboratory Test						
Hemoglobin (g/dL)	0.246 (0.108–0.560)	0.0008	0.366 (0.182–0.738)	0.0049	0.202 (0.083–0.492)	0.0004
Hematocrit (%)	0.658 (0.510–0.851)	0.0014	0.697 (0.545–0.890)	0.0039	0.619 (0.470–0.814)	0.0006
Serum iron (mcg/dL)	0.996 (0.983–1.010)	0.5992	1.004 (0.991–1.018)	0.5374	0.999 (0.986–1.012)	0.8661
Iron saturation (%)	1.000 (0.956–1.046)	0.9945	1.029 (0.982–1.078)	0.2260	1.005 (0.962–1.051)	0.8146
Ferritin (ng/mL)	0.994 (0.979–1.009)	0.4	0.999 (0.985–1.014)	0.8979	0.992 (0.977–1.007)	0.2888
Echocardiography						
LVEF (%)	1.067 (0.958–1.188)	0.2370	1.130 (0.995–1.283)	0.0593	1.101 (0.984–1.232)	0.0942
LVEDD (mm)	0.982 (0.863–1.117)	0.7835	0.904 (0.781–1.046)	0.1738	0.955 (0.839–1.086)	0.4789
LVESD (mm)	0.994 (0.891–1.110)	0.9210	0.944 (0.839–1.062)	0.3369	0.974 (0.873–1.086)	0.6326
IVSd (mm)	0.994 (0.681–1.451)	0.9761	1.050 (0.710–1.552)	0.8067	0.932 (0.642–1.354)	0.7131
IVSs (mm)	1.009 (0.757–1.347)	0.9495	0.932 (0.687–1.264)	0.6495	0.971 (0.730–1.291)	0.8401
LVPWd (mm)	1.034 (0.689–1.550)	0.8719	1.032 (0.678–1.570)	0.8844	1.026 (0.689–1.528)	0.9005
LVPWs (mm)	1.017 (0.819–1.263)	0.8758	1.141 (0.893–1.457)	0.2906	1.030 (0.832–1.276)	0.7832
Mitral E (m/s)	1.117 (0.794–1.571)	0.5270	0.757 (0.519–1.105)	0.1493	0.927 (0.662–1.298)	0.6593
Mitral A (m/s)	0.994 (0.636–1.555)	0.9797	0.926 (0.577–1.485)	0.7497	0.961 (0.618–1.495)	0.8603
E/A	0.989 (0.895–1.093)	0.8320	0.944 (0.849–1.051)	0.2930	0.959 (0.868–1.059)	0.4066
Mitral E′ (m/s)	1.422 (0.324–6.247)	0.6407	0.507 (0.108–2.374)	0.3881	1.212 (0.285–5.149)	0.7945
Mitral A′ (m/s)	1.593 (0.587–4.323)	0.3605	2.078 (0.484–8.932)	0.3255	1.698 (0.534–5.404)	0.37
Mitral S′ (m/s)	0.662 (0.176–2.490)	0.5420	0.487 (0.057–4.182)	0.5119	0.671 (0.198–2.277)	0.5225
Mitral E/E′	0.983 (0.940–1.029)	0.4626	0.966 (0.918–1.017)	0.1876	0.968 (0.924–1.015)	0.1810
Septal E′ (m/s)	1.098 (0.121–9.927)	0.9338	1.211 (0.124–11.844)	0.8695	1.079 (0.124–9.405)	0.9451
Septal A′ (m/s)	0.335 (0.006–19.32)	0.5965	0.145 (0.002–12.897)	0.3990	0.260 (0.004–16.351)	0.5241
Septal S′ (m/s)	2.133 (0.076–59.978)	0.6564	1.037 (0.032–33.406)	0.9838	1.301 (0.049–34.856)	0.8752
Septal E/E′	1.013 (0.981–1.046)	0.4480	0.981 (0.945–1.018)	0.3038	1.001 (0.970–1.033)	0.9567
Tricuspid E′ (m/s)	2.475 (0.347–17.658)	0.3660	2.836 (0.367–21.892)	0.3176	2.469 (0.352–17.325)	0.3631
Tricuspid A′ (m/s)	0.514 (0.055–4.816)	0.5603	0.481 (0.045–5.106)	0.5439	0.568 (0.063–5.119)	0.6143
Tricuspid S′ (m/s)	1.648 (0.062–43.754)	0.7653	2.276 (0.071–72.749)	0.6417	1.374 (0.054–34.790)	0.8473
TAPSE (mm)	0.989 (0.828–1.181)	0.9013	0.910 (0.749–1.106)	0.3440	0.990 (0.831–1.179)	0.9115
PA velocity (m/s)	1.210 (0.064–22.802)	0.8989	0.253 (0.008–7.752)	0.4311	1.000 (0.055–18.083)	>0.9999
IVC diameter (mm)	1.023 (0.871–1.201)	0.7839	0.893 (0.751–1.060)	0.1962	0.998 (0.852–1.169)	0.9807

BMI: body mass index; BSA: body surface area; CI: confidence interval; CMR: cardiac magnetic resonance imaging; IVC: inferior vena cava; IVSd: interventricular septal thickness at diastole; IVSs: interventricular septal thickness at systole; LVEDD: left ventricular end-diastolic dimension; LVEDV: left ventricular end-diastolic volume; LVEF: left ventricular ejection fraction; LVPWd: left ventricular posterior wall thickness at diastole; LVPWs: left ventricular posterior wall thickness at diastole; OR: odds ratio; PA: pulmonary artery; TAPSE: tricuspid annular plane systolic excursion.

**Table 6:** Multivariable logistic regression analyses of myocardial abnormalities on CMR.

	Myocardial Edema on CMR		Myocardial Injury on CMR		Myocardial Edema or Injury (Abnormality) on CMR	
	OR (95% CI)	*p*-Value	OR (95% CI)	*p*-Value	OR (95% CI)	*p*-Value
Age (years)	1.495 (1.031–2.168)	0.0340	1.392 (0.976–1.985)	0.0679	1.573 (1.066–2.321)	0.0224
Female	10.190 (2.347–44.252)	0.0019	7.741 (1.786–33.547)	0.0062	14.323 (3.188–64.357)	0.0005
BMI	0.855 (0.706–1.035)	0.1083	0.950 (0.794–1.136)	0.5750	0.853 (0.703–1.035)	0.1068

BMI: body mass index; CI: confidence interval; CMR: cardiac magnetic resonance imaging; OR: odds ratio.

## Data Availability

All datasets generated or analyzed during the current study are available from the corresponding author on reasonable request.

## References

[1] Sharma O., Sultan A.A., Ding H., Triggle C.R. (2020). A review of the progress and challenges of developing a vaccine for COVID-19. Front. Immunol..

[2] Das B.B., Kohli U., Ramachandran P., Nguyen H.H., Greil G., Hussain T., Tandon A., Kane C., Avula S., Duru C. (2021). Myopericarditis after messenger RNA coronavirus disease 2019 vaccination in adolescents 12 to 18 years of age. J. Pediatr..

[3] Ambati S., Colon M., Mihic M., Sanchez J., Bakar A. (2021). Acute myopericarditis after COVID-19 vaccine in teenagers. Case Rep. Cardiol..

[4] Patel Y.R., Louis D.W., Atalay M., Agarwal S., Shah N.R. (2021). Cardiovascular magnetic resonance findings in young adult patients with acute myocarditis following mrna COVID-19 vaccination: A case series. J. Cardiovasc. Magn. Reason..

[5] Dionne A., Sperotto F., Chamberlain S., Baker A.L., Powell A.J., Prakash A., Castellanos D.A., Saleeb S.F., de Ferranti S.D., Newburger J.W. (2021). Association of myocarditis with bnt162b2 messenger rna COVID-19 vaccine in a case series of children. JAMA Cardiol..

[6] Siripanthong B., Nazarian S., Muser D., Deo R., Santangeli P., Khanji M.Y., Cooper L.T., Chahal C.A.A. (2020). Recognizing COVID-19-related myocarditis: The possible pathophysiology and proposed guideline for diagnosis and management. Heart Rhythm..

[7] Qian Z., Travanty E.A., Oko L., Edeen K., Berglund A., Wang J., Ito Y., Holmes K.V., Mason R.J. (2013). Innate immune response of human alveolar type ii cells infected with severe acute respiratory syndrome-coronavirus. Am. J. Respir. Cell Mol. Biol..

[8] Goulter A.B., Goddard M.J., Allen J.C., Clark K.L. (2004). Ace2 gene expression is up-regulated in the human failing heart. BMC Med..

[9] Guo J., Wei X., Li Q., Li L., Yang Z., Shi Y., Qin Y., Zhang X., Wang X., Zhi X. (2020). Single-cell rna analysis on ace2 expression provides insights into SARS-CoV-2 potential entry into the bloodstream and heart injury. J. Cell Physiol..

[10] Weiss S.R. (2020). Forty years with coronaviruses. J. Exp. Med..

[11] Cooper L.T. (2009). Myocarditis. N. Engl. J. Med..

[12] Sagar S., Liu P.P., Cooper L.T. (2012). Myocarditis. Lancet.

[13] Park J., Brekke D.R., Bratincsak A. (2022). Self-limited myocarditis presenting with chest pain and st segment elevation in adolescents after vaccination with the bnt162b2 mrna vaccine. Cardiol. Young.

[14] Ferreira V.M., Schulz-Menger J., Holmvang G., Kramer C.M., Carbone I., Sechtem U., Kindermann I., Gutberlet M., Cooper L.T., Liu P. (2018). Cardiovascular magnetic resonance in nonischemic myocardial inflammation: Expert recommendations. J. Am. Coll. Cardiol..

[15] Kim P.K., Hong Y.J., Im D.J., Suh Y.J., Park C.H., Kim J.Y., Chang S., Lee H.J., Hur J., Kim Y.J. (2017). Myocardial t1 and t2 mapping: Techniques and clinical applications. Korean J. Radiol..

[16] Truong D.T., Dionne A., Muniz J.C., McHugh K.E., Portman M.A., Lambert L.M., Thacker D., Elias M.D., Li J.S., Toro-Salazar O.H. (2022). Clinically suspected myocarditis temporally related to COVID-19 vaccination in adolescents and young adults: Suspected myocarditis after COVID-19 vaccination. Circulation.

[17] Caforio A.L., Pankuweit S., Arbustini E., Basso C., Gimeno-Blanes J., Felix S.B., Fu M., Helio T., Heymans S., Jahns R. (2013). Current state of knowledge on aetiology, diagnosis, management, and therapy of myocarditis: A position statement of the european society of cardiology working group on myocardial and pericardial diseases. Eur. Heart J..

[18] Writing Committee M., Yancy C.W., Jessup M., Bozkurt B., Butler J., Casey D.E., Drazner M.H., Fonarow G.C., Geraci S.A., Horwich T. (2013). 2013 accf/aha guideline for the management of heart failure: A report of the american college of cardiology foundation/american heart association task force on practice guidelines. Circulation.

[19] Law Y.M., Lal A.K., Chen S., Cihakova D., Cooper L.T., Deshpande S., Godown J., Grosse-Wortmann L., Robinson J.D., Towbin J.A. (2021). Diagnosis and management of myocarditis in children: A scientific statement from the american heart association. Circulation.

[20] Oster M.E., Shay D.K., Su J.R., Gee J., Creech C.B., Broder K.R., Edwards K., Soslow J.H., Dendy J.M., Schlaudecker E. (2022). Myocarditis cases reported after mrna-based COVID-19 vaccination in the us from December 2020 to August 2021. JAMA.

[21] Sachdeva S., Song X., Dham N., Heath D.M., DeBiasi R.L. (2015). Analysis of clinical parameters and cardiac magnetic resonance imaging as predictors of outcome in pediatric myocarditis. Am. J. Cardiol..

[22] Luetkens J.A., Faron A., Isaak A., Dabir D., Kuetting D., Feisst A., Schmeel F.C., Sprinkart A.M., Thomas D. (2019). Comparison of original and 2018 lake louise criteria for diagnosis of acute myocarditis: Results of a validation cohort. Radiol. Cardiothorac. Imaging.

[23] Luetkens J.A., Doerner J., Thomas D.K., Dabir D., Gieseke J., Sprinkart A.M., Fimmers R., Stehning C., Homsi R., Schwab J.O. (2014). Acute myocarditis: Multiparametric cardiac mr imaging. Radiology.

[24] Bohnen S., Radunski U.K., Lund G.K., Ojeda F., Looft Y., Senel M., Radziwolek L., Avanesov M., Tahir E., Stehning C. (2017). Tissue characterization by t1 and t2 mapping cardiovascular magnetic resonance imaging to monitor myocardial inflammation in healing myocarditis. Eur. Heart J. Cardiovasc. Imaging.

[25] Bozkurt B., Kamat I., Hotez P.J. (2021). Myocarditis with COVID-19 mrna vaccines. Circulation.

[26] Gargano J.W., Wallace M., Hadler S.C., Langley G., Su J.R., Oster M.E., Broder K.R., Gee J., Weintraub E., Shimabukuro T. (2021). Use of mrna COVID-19 vaccine after reports of myocarditis among vaccine recipients: Update from the advisory committee on immunization practices—United States, June 2021. MMWR Morb. Mortal. Wkly Rep..

[27] Kaul R., Sreenivasan J., Goel A., Malik A., Bandyopadhyay D., Jin C., Sharma M., Levine A., Pan S., Fuisz A. (2021). Myocarditis following COVID-19 vaccination. Int. J. Cardiol. Heart Vasc..

[28] Jain S.S., Steele J.M., Fonseca B., Huang S., Shah S., Maskatia S.A., Buddhe S., Misra N., Ramachandran P., Gaur L. (2021). COVID-19 vaccination-associated myocarditis in adolescents. Pediatrics.

[29] Isaak A., Feisst A., Luetkens J.A. (2021). Myocarditis following COVID-19 vaccination. Radiology.

[30] Banka P., Robinson J.D., Uppu S.C., Harris M.A., Hasbani K., Lai W.W., Richmond M.E., Fratz S., Jain S., Johnson T.R. (2015). Cardiovascular magnetic resonance techniques and findings in children with myocarditis: A multicenter retrospective study. J. Cardiovasc. Magn. Reason..

[31] Shiyovich A., Witberg G., Aviv Y., Eisen A., Orvin K., Wiessman M., Grinberg T., Porter A., Kornowski R., Hamdan A. (2021). Myocarditis following COVID-19 vaccination: Magnetic resonance imaging study. Eur. Heart J. Cardiovasc. Imaging.

[32] Bellos I., Karageorgiou V., Viskin D. (2022). Myocarditis following mrna COVID-19 vaccination: A pooled analysis. Vaccine.

